# F-actin-based extensions of the head cyst cell adhere to the maturing spermatids to maintain them in a tight bundle and prevent their premature release in *Drosophila *testis

**DOI:** 10.1186/1741-7007-7-19

**Published:** 2009-05-05

**Authors:** Bela S Desai, Seema Shirolikar, Krishanu Ray

**Affiliations:** 1 Department of Biological Sciences, Tata Institute of Fundamental Research, Homi Bhabha Road, Mumbai 400005, India

## Abstract

**Background:**

In *Drosophila*, all the 64 clonally derived spermatocytes differentiate in syncytium inside two somatic-origin cyst cells. They elongate to form slender spermatids, which are individualized and then released into the seminal vesicle. During individualization, differentiating spermatids are organized in a tight bundle inside the cyst, which is expected to play an important role in sperm selection. However, actual significance of this process and its underlying mechanism are unclear.

**Results:**

We show that dynamic F-actin-based processes extend from the head cyst cell at the start of individualization, filling the interstitial space at the rostral ends of the maturing spermatid bundle. In addition to actin, these structures contained lamin, beta-catenin, dynamin, myosin VI and several other filopodial components. Further, pharmacological and genetic analyses showed that cytoskeletal stability and dynamin function are essential for their maintenance. Disruption of these F-actin based processes was associated with spermatid bundle disassembly and premature sperm release inside the testis.

**Conclusion:**

Altogether, our data suggests that the head cyst cell adheres to the maturing spermatid heads through F-actin-based extensions, thus maintaining them in a tight bundle. This is likely to regulate mature sperm release into the seminal vesicle. Overall, this process bears resemblance to mammalian spermiation.

## Background

Spermiogenesis offers a good model for investigating the molecular basis of large-scale cellular morphogenesis and movement. In *Drosophila *, 64 haploid sperm develop from a single gonial precursor through several well-defined morphogenetic steps [1,2]. This entire process happens in three distinct developmental stages: (1) the formation of 64 spermatids from a gonial precursor; (2) the elongation of spermatids from nearly spherical to around 1.8 mm long cells; and (3) the individualization of the elongated spermatids into mature sperm, which then enter the seminal vesicle (SV) [2]. Altogether, it involves large-scale changes in cell shape and internal reorganization [3,4]. Every spermatogonial cell is encapsulated by two somatic-origin cyst cells within the testicular lumen as they form at the testis apex. Subsequent developments occur within this cyst capsule. At the end of the process, the individualized sperm coil up inside the cyst capsule at the base of the testis before entering the SV [4]. Studies in *Drosophila *indicated that defective sperm fail to enter the SV as they fail to remain attached to the head cyst cell during the coiling process [1]. Thus, the dynamics of sperm head attachment to the cyst cell are likely to play a major role in this quality control exercise.

Spermatids grow in an asymmetric manner and the positions of the nuclei define the rostral ends. Subsequently, the nuclei differentiate into needle-shaped structures containing tightly packed DNA and point towards the SV [1,4]. As spermatid elongation proceeds, the rostral ends of the nuclei bundle (NB) advance towards the SV at the basal end of the testis. Then, F-actin-based conical structures called investment cones form around each of the needle-shaped nuclei and shortly thereafter, move down the axoneme at a constant speed [4,5]. This process separately invests each individual spermatid with a plasma membrane, extrudes excess cytoplasm and organelles from the cells, and discharges them as waste bags [3]. Thus individualized, sperm bundles coil up at the base of the testis and then enter the SV [1,4]. F-actin dynamics, myosin VI, dynamin, dynein light chain 1 (DDLC1/LC8) and myosin V [5-9] as well as several pro-apoptotic proteins [10,11] are involved in investment cone assembly and the sperm individualization process. The cyst cells also differentiate along with the spermatids. Two cyst cells of different morphological features are found to encapsulate the spermatid bundle at the start of individualization. The head cyst cell caps the rostral ends like a lid on a tube and the rest of the spermatids are enclosed within the tail cyst cell [1,4]. However, little is known about the molecular basis of sperm release after individualization.

The sperm release process has been extensively studied in the mammalian system. Seminiferous tubules are the functional units of mammalian spermatogenesis. A layer of somatic-origin Sertoli cells line the tubules and constitutes the blood-testis barrier. The Sertoli cells adhere to each other through sets of tight junctions and desmosome-gap junctions [12,13]. The spermatocytes traverse through these sets of inter-cellular junctions from the basal to the adluminal side as they differentiate (stages 1–7), and complete the differentiation (stages 8–16) while still attached to the adluminal side of the Sertoli cell [13-16]. Then they are released as mature sperm into the lumen. This last stage is called spermiation. Prior to this, each spermatid sheds a residual body containing membrane organelles and cytoskeletal elements, which forms at the junction of the sperm head and flagella [14]. This resembles the shedding of waste bag after individualization in *Drosophila *testis [3]. Spermatids also associate with the Sertoli cells through cell-adhesion complexes. A testes specific adherens junction (AJ) called an ectoplasmic specialization (ES) is formed at the inner side of the Sertoli cell [17]. This contains characteristic hexagonal actin arrays packed between the ER cisterns and the plasma membrane with a dense formation of tubulin fibers adjacent to the ER [18,19]. Such a structure has not been reported in invertebrates [13].

In this paper we report the results of a systematic analysis of the final stages of sperm maturation before their release in *Drosophila *testis. We show that the somatic-origin head cyst cell grows F-actin based membranous projections into the interstitial spaces between the mature spermatid heads at the start of individualization. Immunohistochemical analysis showed that these F-actin-rich processes contained markers of filopodia and also proteins found in the AJ. Pharmacological manipulations of the F-actin and microtubule dynamics further revealed that these structures are dynamic and are involved in maintaining mature spermatids in a tight parallel bundle. Finally, a genetic screen identified that *shibire *(dynamin) function is essential to maintain the integrity of these F-actin-based structures and the sperm bundle at the final stage of maturation. Altogether our data provide an initial set of descriptions for further cellular and molecular analysis of spermiation in *Drosophila *.

## Results

### F-actin-based membranous extensions of the head cyst cell cover the sperm heads after individualization

**Figure 1:**
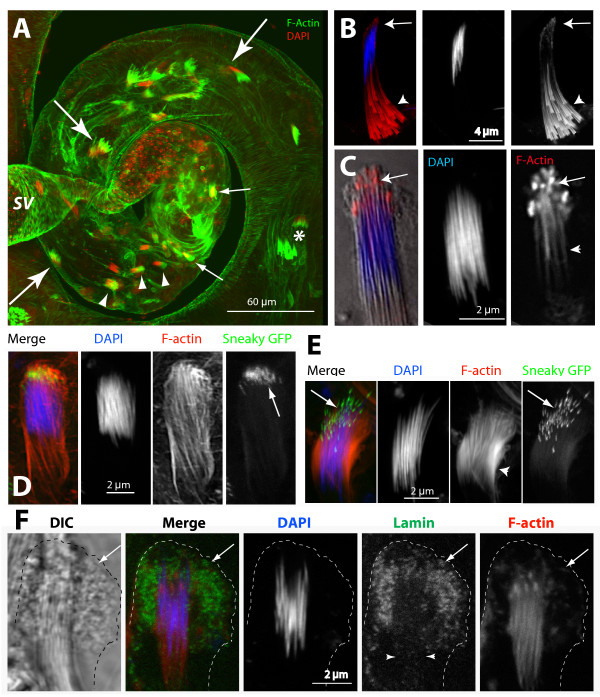
**F-actin-rich structures cap the mature nuclei at the beginning of sperm individualization**. (A) Wild-type testis stained with fluorescein isothiocyanate (FITC):phalloidin (green) and 4',6-diamidino-2-phenylindole (DAPI) (red), respectively. Spermatid NBs associated with investment cones (*), at the early (arrows) and intermediate (arrowheads) stages of individualization, and of the coiled-up stages (fine arrows) are marked. Rostral ends of the NBs moved very slowly towards the SV; see Additional files 1 and 2. (B), (C) DAPI (blue) and rhodamine isothiocyanate (RITC):phalloidin (red) stained isolated cysts show F-actin organization at the rostral ends of spermatids during individualization. (B) Arrow indicates F-actin (red) accumulation at the rostral ends of spermatids at the beginning of the investment cone (arrowhead) assembly. (C) Overlay of the differential interference contrast (DIC) picture (gray scale) of the isolated NB containing the needle shaped nuclei (blue) and the F-actin (arrows) cap at the rostral ends. (D), (E) Isolated cysts from *sneaky-GFP *(green) testis strained as above. (D) NB of a post individualized spermatids. Actin caps appeared around the acrosomes marked by sneaky-GFP (arrows). (E) NB of a relatively later stage bundle (post coiling) found at the testis base. F-actin staining disappeared from the rostral ends but increased laterally (arrowhead). This is presumed to be the penultimate stage before the sperm is released. (F) Combined DIC (Grey) and epifluorescence image of the head cyst cell and associated spermatids stained with anti-Lamin (Dm0) (green), DAPI (blue) and RITC: phalloidin (red). The cell perimeter (broken line) and Lamin-rich membrane folds inside the cell (arrows) are marked. Lamin also localized along the F-actin extensions (arrowheads).

An adult testis contains multiple cysts at different stages of differentiation. They are recognized by the nuclear morphology of the spermatids and the relative positions in the testis. For instance, the needle-shaped nuclei of the elongated spermatids are loosely organized in the cyst (*, Figure 1A, Additional files 1 and 2) at the start of individualization and these are generally found at about 250 μm from the base. The F-actin-rich investment cones (also known as F-actin cones) form around each nucleus during the individualization stage and move towards the caudal ends in synchrony [1,3,5,6]. The NBs of the individualizing spermatids (arrowheads, Figure 1A) are found in a region 200–255 μm from the testis base (Figure 1A), and they move further towards the SV in subsequent developmental stages (fine arrows, Figure 1A) until the mature sperm enter the SV. The entire process was estimated to take nearly 20 hours [1,3]. Therefore, the rostral ends of the maturing spermatid bundles are likely to traverse around 250 μm to the SV in as much time or longer.

Additional F-actin accumulations were observed at the rostral tips of the NBs (arrows, Figure 1B) at the start of individualization. Subsequently, the F-actin grew as cap-like structures around the spermatid nuclei during individualization (Figure 1C). The F-actin densities were found around rostral tips of individual nuclei and acrosomes of an NB (Figure 1D), marked by the sneaky-GFP [20]. At a later stage when the individualized and mature sperm coiled up inside the cyst, the F-actin densities were mostly found around the lateral sides of the nuclei (arrowhead, Figure 1E). This is likely to correspond to a stage when sperm were about to be released as both the acrosomes and the NB appeared unpacked (arrow, Figure 1E). These F-actin based structures will be referred as 'actin caps' in the subsequent discussion. The actin caps were also observed to form inside the head cyst cell covering the rostral ends of maturing spermatid bundles (Figure 1F) and occasionally 'empty' actin caps not associated with the NB were also observed inside the testis. These observations raised an obvious question about the cellular origin of the actin caps.

**Figure 2:**
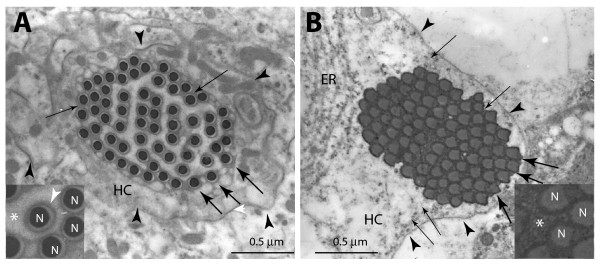
**Transmission electron microscopy images of transverse sections of mature sperm heads inside a cyst at the base of a wild-type testis (magnification 28,000×)**. (A) Cross-section view of mature and individualized sperm nuclei (arrows) inside the head cyst cell (HC, arrowheads). The stage is determined according to an earlier description [4]. Inset shows enlarged view of the nuclei (N) tightly invested with plasma membrane (white arrowhead). The nuclei are embedded in an electron dense material (fine arrows) and membranous projections filled the interstitial space (marked with *). (B) Nuclei (arrows) of post-individualized spermatids inside the head cyst cell are tightly packed with electron dense material around them (fine arrows). They were also placed at one side of the HC. Inset shows 10× enlarged view of part of the bundle. The interstitial space (*) is packed with electron dense material.

Transmission electron microscopy (TEM) studies further showed that the nuclei of the 'individualized' spermatids were tightly invested with plasma membrane (arrows, Figure 2A) and contained very little cytoplasm. They were embedded into the head cyst cell (HC, Figure 2A) with electron dense material around (fine arrows, Figure 2A; see also the inset). An earlier study reported that membrane bound projections containing microfilaments are extended from the head cyst cell and interspersed between the sperm heads after individualization [4]. We also found some tightly packed NBs (arrows, Figure 2B) with dense material around them near the head cyst cell perimeter (fine arrows, Figure 2B; see also the inset). Together with the previous results this suggested that the actin caps are likely to form inside the head cyst cells and therefore, unlikely to be a part of the spermatids.

**Figure 3:**
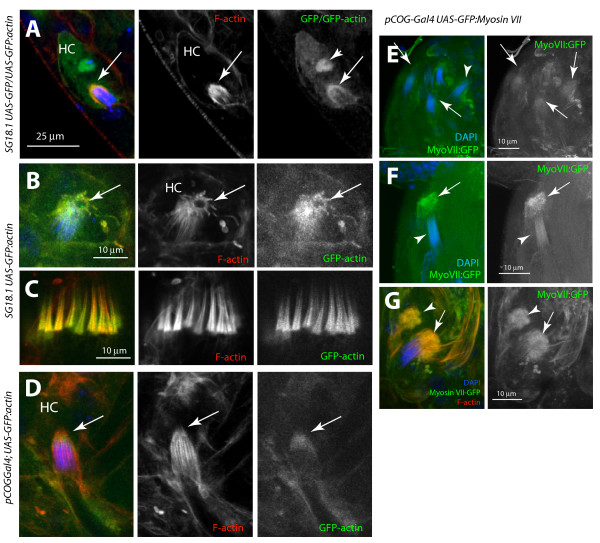
**Actin caps are extended from the head cyst cell**. (A) Optical section from the *SG18.1Gal4 UAS-GFP/UAS-GFP:actin *testes stained with rhodamine isothiocyanate (RITC):phallodin (red) and 4',6-diamidino-2-phenylindole (DAPI) (blue) show GFP and GFP-actin localizations in the head cyst cell nucleus (arrowhead) and in the actin cap (arrows), respectively. (B), (C) A similar staining of the *SG18.1Gal4 UAS-GFP:actin/+ *testis shows prominent GFP:actin localization in the actin cap (arrows, (B)) and also in the investment cones (C). (D) The actin cap (arrows) is also labeled by GFP:actin in *pCOGGal4/Y; UAS-GFP:actin/+ *testis. (E)-(G) Confocal sections from the DAPI (blue) stained *w pCOGGal4 UAS-myosin VII:GFP/Y *testis show myosin-VII:GFP (green) localization around the nuclei bundles (NBs) (arrows, (E)) at the testis base. (F) Higher magnification image of a single NB show prominent myosin VII:GFP localization along the NB (arrowheads) and also in the head cyst cell (arrows). (G) RITC:phalloidin (red) and DAPI (blue) staining further revealed that the myosin VII:GFP is enriched in the actin cap (arrows). The arrowheads indicate an empty actin cap. (See Additional file 3 for a detailed analysis of expression pattern in testes.)

To further establish this point we used two Gal4 stocks, (a) *SG18.1Gal4 *and (b) *pCOGGal4 *, which were found to express in the head cyst cells during the final stages of sperm maturation (Additional file 3). Although the *SG18.1Gal4 *expression was also found in the spermatocytes at an earlier stage, the *pCOGGal4 *expression was limited to the cyst cells from the very beginning (Additional file 3). The expression of *UAS-actin:GFP *in *SG18.1Gal4 *background marked the actin caps (arrows, Figures 3A and 3B) and the F-actin cones (Figure 3C). However, only the actin caps were marked in the *pCOGGal4/Y; UAS-actin:GFP/+ *testis (arrows, Figure 3D). Furthermore, the recombinant *myosin-VII:GFP *expression in the *w pCOGGal4 UAS-ck:GFP *stock clearly marked the actin caps at the rostral ends of mature NBs (arrows, Figure 3E–G). These observations confirmed that the actin caps are indeed formed inside the head cystcells. In addition, the actin caps were always found attached with the spermatid heads in squash preparations. This suggested that these F-actin-based extensions of the head cyst cell are likely to adhere to the maturing sperm heads. However, TEM studies did not reveal obvious cellular junctions between the sperm heads and the cyst cells.

### Cell adhesion proteins along with certain filopodial components are enriched in the actin caps

**Figure 4:**
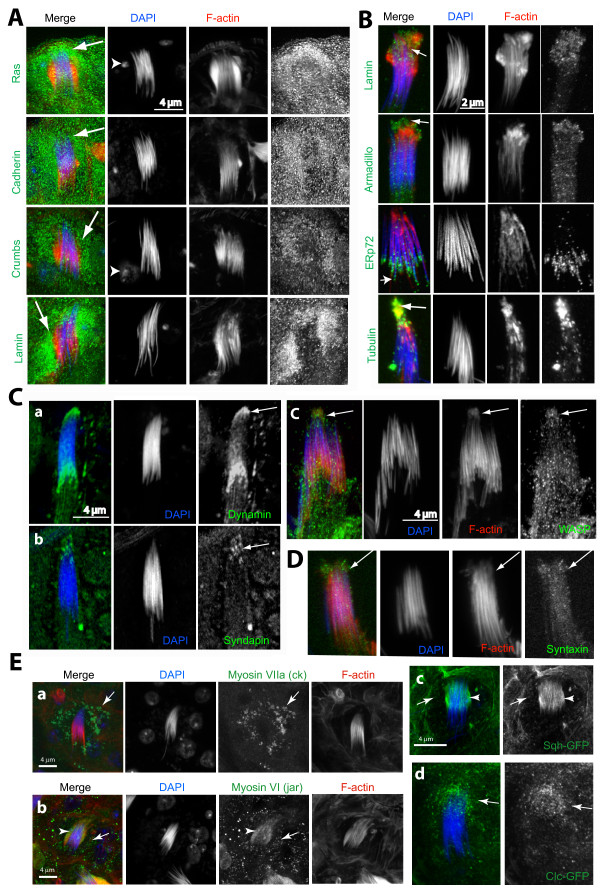
**Immunohistochemical characterization of the actincap**. (A) Confocal images of head cyst-cells in intact testis containing mature and individualized sperm nuclei from the basal regions of testes, stained with: ras, DE-cadherin, crumbs and lamin antisera (all in green), as well as rhodamine isothiocyanate (RITC): phalloidin (red) and 4',6-diamidino-2-phenylindole (DAPI) (blue). Arrows indicate staining in the membranous folds inside the head cyst cell and arrowheads indicate the head cyst cell nuclei. (B) Isolated mature spermatids without the head cyst cells as found in the testis squash preparations stained with lamin (Dm0), armadillo, tubulin and ERp72 antisera (indicated at the left side panel of each set). (C), (D) Similar preparations stained with the dynamin (a), syndapin (b) and WASP (c) as well as (D) Syntaxin (green). Arrows indicate positions of the actin caps. (E) Head cyst cells inside testis immunostained with the (a) ck/myosin VII and (b) jar/myosin VI, respectively, or expressing (c) the *sqh-GFP *and (d) the clathrin light chain:GFP (*clc-GFP *), respectively. (a) ck (green) staining (arrows) coincided with the membranous fold and (b) anti-jar stained the actin cap (arrows) as well as a few punctate spots in the cytoplasm (arrowheads). (c) The sqh-GFP localized along the actin caps (arrowheads) and in punctate spots (arrows) in the cytoplasm. (d) *clc-GFP *mostly localized in the punctate spots (arrows) in the cytoplasm. The *sqh-GFP *transgene is expressed through its own promoter in *sqh *homozygous mutant background and the *UAS-clc-GFP *is expressed by using the *actin5CGal4 *(see Additional file 6 for details).

Therefore, to further understand the role of the actin caps, we decided to characterize its molecular composition. Regulation of actin dynamics and AJs are known to play crucial roles in epithelial cell morphogenesis and movement during development. In addition, the basolateral protrusive activity of these cells that penetrate into the neighboring cells to promote the formation of adhesive contacts is suggested to involve actin [21]. Immunostaining revealed that prominent AJ markers such as DE-cadherin and crumbs were enriched in the head cyst cells (arrows, Figure 4A). The *Drosophila *lamin (Dm0), armadillo (beta-catenin), tubulin and ERp72 were also enriched at the actin caps (arrows, Figure 4B). ERp72, which seemed to be enriched more caudally than the others, is an ER resident protein found in testes specific AJs [22,23]. This suggested that actin caps could adhere to the sperm heads through AJs. Actin-based membrane extensions such as filopodia and invadopodia are also known to require dynamin, syndapin and WASP [19,24-27]. These were also associated with the actin caps (arrows, Figure 4C, a, b and 4C, c). In addition, syntaxin [28], a t-SNARE involved in membrane fusion events, was present along the actin cap extensions (arrows, Figure 4D). Thus, actin caps contained proteins characteristic of both filopodia and AJs. They are likely to adhere to sperm heads through beta-catenin, which is an integral component of AJ in the epithelial cells [29] and testes [19].

Filopodia and pseudopodia require the functions of different unconventional myosins [26,30-33]. We found that both myosin VII (ck) and myosin VI (jar) were present in punctate spots in the head cyst cell cytoplasm (arrows, Figure 4E) and jar/myosin VI was enriched along the actin cap extensions (arrowheads, Figure 4E, b). In addition, expression of the recombinant myosin II regulatory light chain-GFP (sqh-GFP) [34] also marked the actin caps (arrowheads, Figure 4E, c). Myosin VI is known to stabilize F-actin bundles in the microvilli and the stereocilia [33], and both myosin II and VIIa were shown to maintain vesicular traffic into the microvilli projections [33,35]. In *Drosophila *ovary, myosin VI was localized in the membrane protrusions at the leading edge of migrating border cells [36], and at the investment cones in the testis [6]. Therefore, the actin caps appeared to be filopodia-like projections of the head cyst cells maintained by myosin-dependent vesicular traffic. However, we noted that unlike the ck:GFP, the ck antibody did not label the actin cap extensions. The ck antibody is raised against the stalk domain of the predicted myosin VII ORF [37], which is likely to be involved in homodimerization of the motor subunits and also in binding other accessory subunits. Therefore, unlike the ck:GFP, the antibody could only label myosin VII (ck) in the tissue if the epitope is exposed. Furthermore, the ck:GFP transgene is ectopically expressed using a non-homologues promoter and this could also alter its subcellular localization in the cell. All of these could account for the apparent differences in the subcellular staining patterns of ck in the head cyst cell.

A large number of vesicles were reported to accumulate inside the head cyst cell around the rostral tips of the embedded sperm heads [4]. This was also revealed in the DIC images of the isolated head cyst cells. The punctate distributions of myosins in this region further suggested that these motors might be involved in vesicle transport into the actin cap projections. Myosin VI was known to associate with the clathrin-coated pits inside the cell during endocytosis as well as with dynamic membrane ruffles in the migrating epithelial cells [33]. We found that the clathrin light chain-GFP (clc-GFP) was enriched in punctate spots around the actin caps (arrows, Figure 4E, d) in the *w; UAS-clc-GFP Actin5cGal4 *testis. Clathrin is involved in coated vesicle assembly [38] and plays an important role in dynamin-mediated vesiculation inside the cell and the clc is an integral part of the clathrin complex. Therefore, this observation further supported the hypothesis that vesicular traffic from the head cyst cells could supply membrane to the actin cap projections. Altogether the immunolocalization results indicated that actin caps are filopodia-like extensions and likely to attach to the sperm heads during individualization.

### F-actin and microtubule stability are essential for actin cap assembly and maintenance

**Figure 5:**
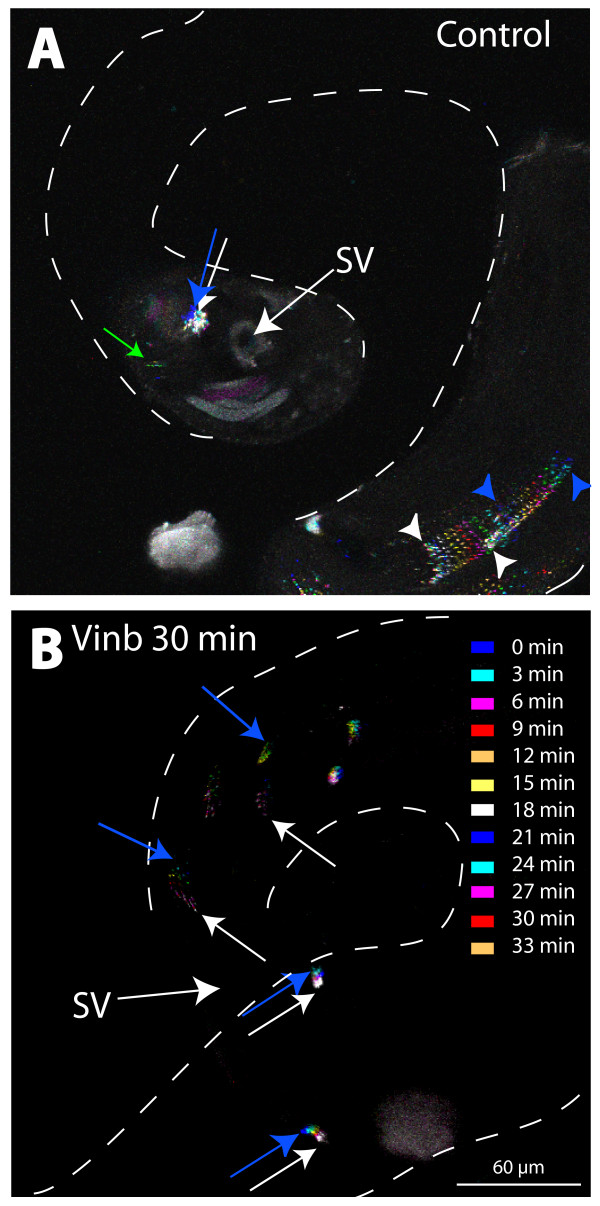
**Mature spermatid heads remain in a tight bundle attached to the testis wall during the coiling stage**. (A), (B) Sets of time lapse images of the testis base, expressing the sneaky-GFP transgene [20], were projected together to show the relative movement of the acrosome bundles of a cyst inside the testis. Each frame is labeled with a specific false color as per the list shown in (B). The blue arrows and arrowheads point to the positions of acrosome bundles in the first frame while the white arrows and arrowheads indicate the final position. The arrows indicate the compacted set, which is likely to belong to the post-individualization stages, while the arrowheads indicate the acrosomes of the elongating/pre-individualization stage spermatids. (A) The compacted acrosome bundle (arrows) found near the base of the testis remain confined in the region as indicated by the positions of the blue and white arrow. Some acrosomes (green arrow) are occasionally found to move away from the bundle. This is considered to belong to the defective sperm that are lost during coiling. In comparison the acrosomes of the elongating spermatids (arrowheads) are loosely organized and move rapidly towards the testis base as evident from the positions of the blue and white arrowheads. (B) The mobility of acrosome bundles (blue and white arrows) near the base of the testis increased after 30 minutes of 5 μM vinblastine (*vinb *) treatment. (See Additional files 2 and 4 for details.)

Spermatids are maintained in a tight bundle inside the cyst during individualization and this is predicted to play a critical role in selecting out the improperly developed sperm [4]. Live analysis of acrosome movements inside testis by using the sneaky-GFP [20] further showed that the acrosome movements slow down after they are packed (arrows, Figure 5A, Additional files 2 and 4). This coincided with actin cap formation and, thus, suggested that the actin caps are required to maintain the sperm heads in a tight bundle during individualization. Indeed we noticed that few mature nuclei were left out in every NB in almost all wild-type testes during the coiling stage. Both F-actin and microtubule dynamics play important roles in the filopodia/pseudopodia assembly and maintenance [39-41]. Latrunculin B (lat B) binds to actin monomers and prevents their polymerization and the addition of lat B to cultured cells leads to major disruption of actin cytoskeleton [41]. Similarly, treatments with vinblastine (vinb) leads to microtubule depolymerization in the tissue [42,43]. Therefore, to determine the role of the cytoskeletal elements in actin caps, we treated intact testis with these reagents.

**Figure 6:**
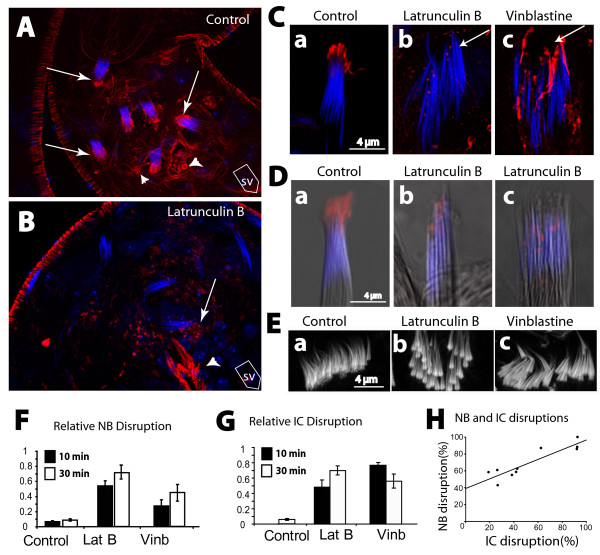
**F-actin and microtubule maintain the actin cap, essential to maintain the maturing spermatids in a tight bundle**. (A), (B) Wild-type testis stained with 4',6-diamidino-2-phenylindole (DAPI) (blue) and rhodamine isothiocyanate (RITC):phalloidin (red) after 30 minutes in PBS containing, (A) 8 μM DMSO (control), or, (B) 30 μM latrunculin B (lat B). Arrows indicate NBs of the mature sperm associated with the actin caps and arrowheads indicate residual caps left behind after sperm release. SV indicates the direction of the seminal vesicle. (C), (D) Isolated cysts stained with DAPI (blue) and RITC:phalloidin (red) after 30 minutes of incubation in PBS containing either, (a) 8 μM DMSO (control), (b) 30 μM lat B, or (c) 5 μM *vinb *as indicated at the top of the panels. Corresponding DIC images were overlaid in grey scale (D) to indicate the contours of the sperm heads. (E) F-actin cones of ICs from intact testis treated with (a) DMSO (control), (b) lat B and (c) *vinb *, respectively. (F), (G) Percentage NB (F) and IC (G) disruptions, after 10 (solid bars) and 30 (open bars) minutes of treatments presented as histograms with +S.E.M., and n > 6 for individual plots. (H) The points in the plot represent statistical correlations between the IC and the NB disruption indices within individual testes after 30 minutes of the lat B treatments. A straight line fitted through the points indicates the probability of a simultaneous occurrence of the two events together as estimated from the observed data (see Table 1 and associated legend for detail).

The acrosome bundles were disrupted and the individual acrosomes moved about each other in the bundles after 30 minutes of the 5 μM vinb treatment (arrows, Figure 5B). A similar result was obtained with the 25 μM lat B as well (data not shown). It also caused severe actin cap disruptions (Figure 6A and Table 1) within 10–30 minutes, with visible loss of F-actin (arrows, Figure 6B and 6C). Although the 5 μM vinb treatment did not abolish the F-actin staining, it disrupted both the actin caps and the NBs (arrow, Figure 6D). These observations suggested that the stability of both the microtubule and F-actin cytoskeleton are essential to maintain the actin caps and this appeared to disrupt the NBs. In addition, the pharmacological treatments also disrupted the F-actin cone organization in the individualization complex (IC) (Figure 6E), although the staining at the F-actin cones was not visibly altered. Finally, a quantification study showed significant increase in the disrupted NB and IC populations after the drug treatments (Figure 6F and 6G), and the disruption indices were strongly correlated in each testis (Figure 6H). The conjugated NB and IC disruptions reflected disorganizations of the spermatid bundles inside the cysts and the rapid manifestation of the defect further suggested that the association between the head cyst cell and the spermatids are dynamic. F-actin disruption in isolated testis of adult moths was shown to block sperm release and spermatid bundle disorganization [44] and studies in mammalian testis showed that the F-actin stability in the apical ES is essential to maintaining association between developing spermatids and Sertoli cells [12].

**Table 1:** Relative NB and IC disruptions in individual testis after 30 minutes of *lat B *(25 μM) treatment.

Testes number	NB disruption (%)	IC disruption (%)
1	92.6	88.2
2	26.7	43.0
3	19.0	58.3
4	26.3	61.0
5	42.9	62.5
6	92.9	100
7	38.5	55.0
8	41.9	58.6
9	92.3	85.7
10	62.5	87.0

The percentage values are calculated with respect to the total number of NBs and ICs in the same testis. A statistical cross-correlation plot of this data is shown in Figure 6G. Such disruptions were not observed in the control testes treated with 8 μM DMSO alone.

### shibire/dynamin is essential for maintaining the actin caps and spermatid bundles

The association between head cyst cells and spermatids was predicted to play an important role in spermiation [4]. The above results indicated that the head cyst cells are likely to adhere to the maturing sperm heads through F-actin-rich projections containing several filopodial markers. Dynamin plays a key role in membrane reorganization process and our immunolocalization data suggested that it could play an important role in actin cap assembly or maintenance as well. The *shibire *gene codes for a dynamin homologue in *Drosophila *, which is shown to play an important role in endocytosis [45] and various other cellular functions [46]. Previous studies have also shown that *shi *^*ts1 *^(a conditional mutant allele of *shibire *) hemizygous flies rapidly paralyze within a few minutes at non-permissive temperatures [47]. Thus, the conditional *shi *^*ts1 *^alleles provided a good tool to further test the role of dynamin in actin caps and the latter's role in sperm maturation/release.

**Figure 7:**
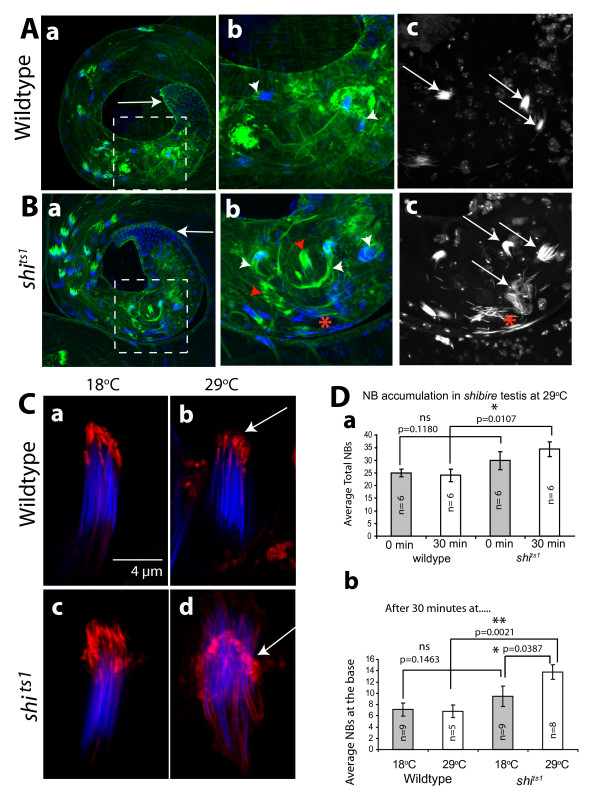
**Actin caps and NBs are disrupted in hemizygous *shibire *mutant testes after heat pulse**. (A), (B) Wild type (A) and hemizygous *shi *^*ts1 *^mutant (B) testes labeled with FITC:phalloidin (green) and 4',6-diamidino-2-phenylindole (DAPI) (blue) after 30 minutes at 29°C. Regions populated by the coiled-up sperm bundles are marked by the dotted lines. Note that the base (arrows) is slightly enlarged in *shi *^*ts1 *^hemizygous testis. Figures (b), (c) of (A) as well as (B) are enlarged views of the regions marked in (a) of (A), and (a) of (B), respectively. The figures in the (c) of A and (B) indicate the DAPI labeled NBs. The white arrowheads indicate actin caps and arrows indicate the NBs in respective figures. The red star indicates a highly disrupted NB and red arrowhead indicates abnormal actin caps in figures (b) and (c) of (B). (C) Isolated cysts stained with RITC: phalloidin (red) and DAPI (blue) from (a, b) wild type control and (c, d) the *shi *^*ts1 *^hemizygous mutant testes before and after 30 minutes at 29°C. Arrows indicate actin caps in figures (c) and (d). (D) Histograms indicate average NBs in a testis in wild type and *shi *^*ts1 *^adults after 30 minutes of incubation at 18°C (grey filled bars) and at 29°C (open bars), respectively. The error bars indicate ± S. E. M. The number of specimen (n) for each bar and the pair wise test of significance (p values) are indicated on each figure. The non-significant (ns), significant (*) and very significant (**) differences are indicated on each set of bars linked by the horizontal lines.

Testes from the *shi *^*ts1 *^hemizygous males grown at 18°C had no apparent defect in NB organization and actin cap morphology (Figure 7A). However, some of the actin caps (white arrowheads, Figure 7B, b) and a few NBs (red *, Figure 7B, c) appeared disrupted after 30 minutes at 29°C. In addition, a few actin caps were found without the associated nuclei (red arrowheads, Figure 7B, b). Interestingly, F-actin levels in the disrupted actin caps were not reduced (Figure 7C), indicating that *shibire/dynamin *functions were required to maintain the attachments to sperm heads. In addition, there was a small but significant increase in the numbers of mature NBs at the testis base after the heat treatment (Figure 7D). Since the gonial precursors form at an hourly rate, cysts are expected to mature at the same rate. Therefore, the small increase in the NBs after a 30 minute pulse is quite significant. This indicated that *shibire *could regulate premature sperm release. This would also suggest that the sperm maturation process is very dynamic and the *shibire/dynamin *function is constitutively required to maintain the association between the sperm heads and the head cyst cell. However, this failed to resolve whether the shibire/dynamin acts at the actin caps.

### shibire/dynamin function is required in the head cyst cells to maintain the actin caps and the sperm head bundles

**Figure 8:**
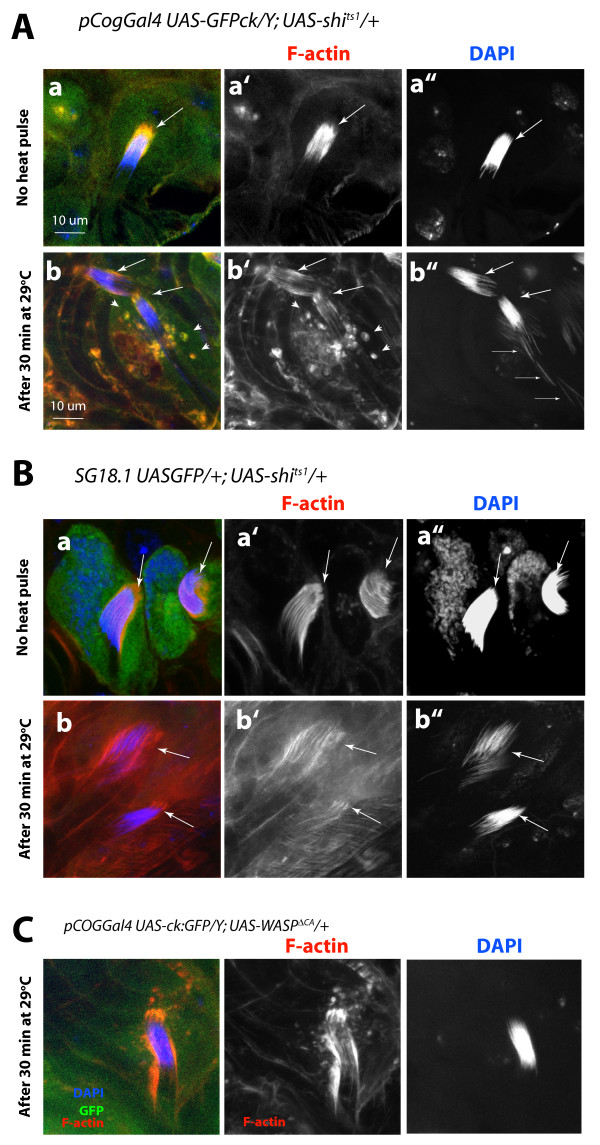
**Transient disruption of the *shibire *function in the head cyst cell caused actin cap and nuclei bundle disruptions**. The *UAS-shi *^*ts1 *^expression in the head cyst cells due to the (A) *pCOGGal4 *and (B)*SG18.1Gal4 *drivers, respectively, disrupted the actin caps and nuclei bundles (NBs) at non-permissive temperature (29°C). Rhodamine isothiocyanate (RITC):phallidin and 4',6-diamidino-2-phenylindole (DAPI) staining of these testes (a) before and (b) after the heat pulse showed visible disruptions of the actin caps and NBs (arrows). In addition, there was punctate accumulation of F-actin in the head cyst cell cytoplasm (arrowheads) after the heat pulse. Fine arrows indicate mature nuclei separated from the actin caps. This is not found in the wild-type controls treated in a similar manner. The actin cap and the NB morphology remained unaltered even after 30 minutes at 29°C in the *pCOGGal4 UAS-GFPck/Y *and *SG18.1 UAS-GFP/UAS-actin:GFP *testes (data not shown). (C) The *UAS-WASP *^*ΔCA *^expressions by using the *pCOGGal4 *caused no detectable NB disorganization and a mild accumulation F-actin rich spots in the cytoplasm after a 30 minute heat pulse. (See Additional file 5 for further details.)

The ectopic expression of the recombinant *UAS-shi *^*ts1 *^transgene was shown to disrupt the endogenous shibire functions at non-permissive temperature (29°C) in a dose-dependent dominant-negative manner [48]. We used this technique to temporally perturb the *shibire/dynamin *function in the head cyst cells. The *UAS-shi *^*ts1 *^expression in the head cyst cells owing to the *pCOGGal4 *as well as the *SG18.1Gal4 *drivers caused visible actin cap and NB disruptions within 30 minutes at 29°C (Figure 8A and 8B, Additional file 5). In addition, F-actin rich spots appeared to accumulate in the head cyst cell after the heat pulse (arrowheads, Figure 8A and 8B). Same set of *Gal4 *driver stocks were used to drive *UAS-WASP *^*ΔCA *^and *UAS-GFP *expressions in the head cyst cells as controls. The recombinant WASP^ΔCA ^lacks 30 amino acids in the C-terminal part constituting the VCA domain of the wild-type protein and therefore, it cannot bind to Arp2/3 [49]. Its expression in the *Drosophila *myoblasts caused fusion defects [50]. However, expressions of the *UAS-WASP *^*ΔCA *^(Figure 8C) or *UAS-GFP *in the head cyst cells did not disrupt the actin caps even after the heat pulse. These observations further established that the effects observed after the heat pulse due to the ectopic *UAS-shi *^*ts1 *^expression in the head cyst cells are indeed caused by the presence of recombinant shibire(ts1) protein, and further proved that the shibire function is required in the head cyst cells to maintain the actin cap morphology and its attachment with the spermatid heads. Since the shibire antigen along with syndapin is enriched near the actin caps, this could further suggest that dynamin function is required in the actin cap region. Therefore, sihibire/dynamin mediated membrane dynamics in the actin cap region is likely to play an important role in maintaining adhesion between the maturing sperm heads and the head cyst cell, and thus prevent premature release of spermatids inside the testis.

## Discussion

**Figure 9:**
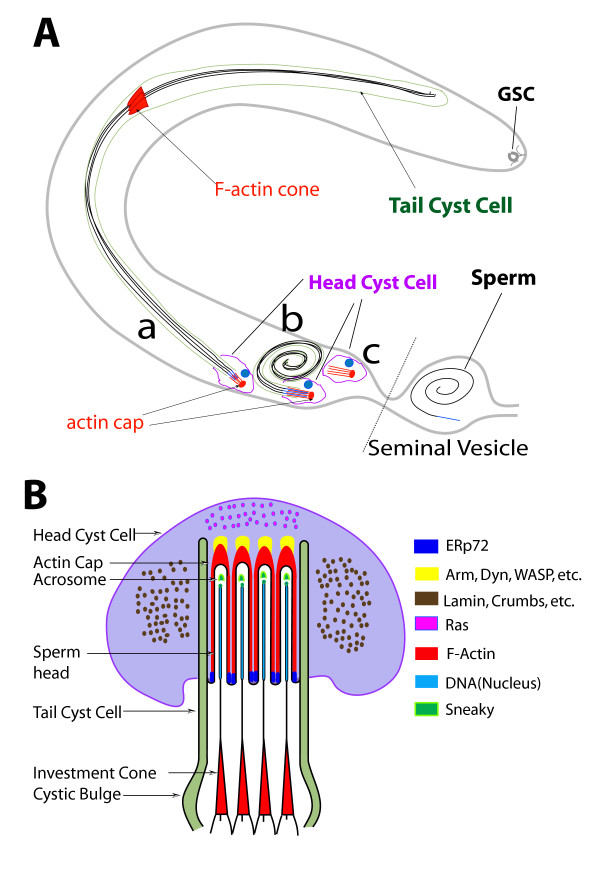
**Anatomical organization and molecular compositions of actin cap structures around the sperm head assembly inside head cyst cell**. (A) Summary of the observations and indication of the positions of (a) the individualization stage, (b) coiled-up stage and (c) empty cysts inside a testis (gray out line). The positions of germ line stem cell (GSC) at the apex and the head and tail cyst cells are marked with distinct colors as indicated in the figure. The axoneme of maturing spermatids (black), the cell nuclei (blue) and major F-actin based structures (red) are highlighted to illustrate the basic observation of this study. The rostral ends of the sperm nuclei moved very slowly towards seminal vesicle during individualization until the mature sperm penetrate the head cyst cell to enter the seminal vesicle. (B) Cross-section view of the relative organizations of actin caps inside head cyst cell and sperm heads during individualization. It is partly adapted from a previous description [1] and updated to summarize the data presented in this report. Only four spermatids are drawn for simplicity in illustrating the organization. The compositions of the different regions are indicated by color coding as shown in the adjacent panel.

In summary, our results highlight the cellular and molecular mechanism of the sperm bundling process in *Drosophila *. Previous anatomical studies in *Drosophila *[4] as well as in several other insects [51,52] established that spermatids are tightly bundled towards the end of differentiation and before their release into the SV. This process is predicted to single out abnormal sperm after individualization and thus acts as a quality control step in spermiogenesis [1,4]. However, the cellular and molecular mechanism underlying this process was unknown. The sperm heads are embedded into the somatic-origin head cyst cell at the start of individualization. We have shown that they are held by the head cyst cell through F-actin-based extensions (Figure 9A), Immunohistochemical characterizations combined with pharmacological interventions showed that these are filopodia-like extensions adherent to the maturing sperm heads (Figure 9B). Finally, molecular genetic analysis suggested that shibire/dynamin function is essential in the head cyst cells to maintain the sperm heads in a bundle and prevent their premature release inside the testis. These observations have several interesting parallels with the spermiation process in the mammalian system as discussed in the following sections.

### Actin caps organize spermatid heads in a tight bundle inside the head cyst cell

Post individualization, all of the 64 clonally derived spermatids are twisted together and the bundle coils up inside the cyst. The head cyst cell attaches to a terminal epithelial cell at the base of the testis, and then the sperm is released into the SV [4]. Our studies identified a specific function of the head cyst cell in the sperm bundling process during the maturation stage. As summarized in Figure 9B, it is found to grow several F-actin-based extensions at the start of individualization that are enriched with proteins found in the filopodia of other cell types [53]. In addition to dynamin, syndapin and WASP, proteins found in the apical ES [54,55] such as ERp72, E-cadherin and beta-catenin are enriched in the actin cap extensions. This suggests that the actin cap extensions are dynamic membrane bound projections and they adhere to the individualized sperm heads while the remaining parts of the spermatids undergo morphogenetic changes during the individualization and coiling stages. Lamin, a protein associated with the F-actin cones during the sperm individualization process [10] was enriched at these structures. This could strengthen the membrane cytoskeleton and provide rigidity to the actin cap extensions. Myosins are essential for the formation of filopodia-like extensions [33] and for cell adhesion [56,57] in several other cell types. Hence, the presence of the myosin II regulatory light chain, Myosin VI and myosin VII suggests frequent vesicular membrane transport into and out of the actin cap projections as well as cross linking of actin filaments to the plasma membrane inside these extensions. The presence of clathrin light chain and syntaxin further pointed towards the existence of dynamic membrane exchange along the actin cap projections. Altogether these immunolocalization studies suggested that actin cap extensions could adhere to the maturing sperm heads during individualization and coiling through a combination of different cellular mechanisms. This is also confirmed by the results of our pharmacological and genetic analyses, which indicates that the actin cap extensions and their interactions with the spermatid heads are dynamic. Altogether, it showed that the differentiating spermatids are tightly attached to the head cyst cell until they are fully mature and suggested that this could play a critical role in preventing premature sperm release inside the testis.

F-actin-based membrane remodeling at filopodia and pseudopodia-like structures is involved in cell migration, axonal growth cone guidance and cell ingestion [58]. In *Drosophila *ovary, the somatic origin 'border cells' migrate through the germ-line 'Nurse cells' by extending similar F-actin-based membranous projections [59]. Actin-based cellular extensions were also known to anchor epithelial cells on the substratum involving beta-catenin and cadherin [36]. White blood cells migrate out of the blood vessel by penetrating the endothelial cell layer through such actin-based membranous projections by a process called transcytosis [16]. *Drosophila *spermatids lose all of their cytoplasmic contents after individualization by the movement of the F-actin-based investment cones through them [3,7]. Hence, the somatic-origin cyst cells that encapsulate these near-mature sperm are expected to play an important role in regulating their release. This is further established by a targeted disruption of *shibire/dynamin *functions in the head cyst cells, which indicate that the maintenance of spermatids in a tight bundle is essential for preventing a premature release. This is also consistent with observations made in the mammalian testis. The Sertoli cells play an important role in the mechanical movement of germ cells from the basal to the adluminal side during their differentiation [12]. This process is aided by the F-actin-rich ES and a variety of different cellular junctions [14-16]. Our results helped to identify the molecular and cellular framework involved in the spermiation process in *Drosophila *. This also provides a useful base for future analysis of sperm release mechanisms.

### Dynamin-mediated membrane remodeling is essential for actin cap maintenance and attachment to sperm nuclei at the terminal stages of maturation

Dynamin is implicated in the assembly and maintenance of F-actin-based membrane ruffles, podosomes and invadopodia [60-63], and a specific dynamin isoform in rat (dyn3) was found to associate with the tubulobulbar junctions around the sperm heads [64,65]. Dynamin is enriched at the actin caps and genetic analysis showed that they are essential for maintaining the sperm bundle inside the cysts. The dynamin requirement appeared to be constitutive as the temporal loss of *shibire/dynamin *function caused actin cap disassembly and NB disorganization. In addition, it caused accumulation of NBs inside the testes within 30 minutes. Thus, dynamin-mediated membrane remodeling at the actin cap region is expected to play a role in sperm bundling and release processes in *Drosophila *, and provided a basis to investigate the interactions between the somatic origin cyst cells and spermatids during the sperm maturation process in *Drosophila*.

## Conclusion

In view of the observations presented here, the sperm maturation process in *Drosophila *resembled the spermatid development inside the Sertoli cell layer in mammalian testis [14]. The mammalian spermatids attach to the Sertoli cell membrane through the apical ES [66]. The F-actin-based ES forms around developing spermatids inside the Sertoli cell [13]. Although electron microscopic studies did not reveal ES-like structures around the sperm heads in *Drosophila *testis, the actin caps contained the essential functional elements of these specialized junctions. It is rich in F-actin, beta-catenin and DE-cadherin, and tightly associated with the spermatid heads during individualization. In addition, we found a second interesting parallel. Mammalian sperm are physically released from the Sertoli cells after the removal of integrin and beta-catenin from the apical ES [67]. A tubulobulbar junction forms inside the Sertoli cells and around the mammalian spermatids [54,55]. Proteins involved in endocytosis, such as dynamin 3 and amphiphysin were enriched at these junctions [64,65,68], and loss of amphiphysin from the tubulobulbar junctions was shown to block sperm release in the knockout mice [68]. We showed that shibire/dynamin functions in the head cyst cells in *Drosophila *testis to maintain the actin cap integrity and sperm heads in a tight bundle. Thus, our observations have the potential to establish *Drosophila *as an attractive model for molecular analysis of spermiation in insects. It has defined an assay to study the role of F-actin-mediated cell adhesion process and can be used to screen for small molecule-based perturbation of the sperm maturation process in the future.

## Methods

### Drosophila stocks and culture conditions

All of the fly stocks used for this study are listed in Additional file 6. They were maintained on standard cornmeal agar medium as described previously [46]. We sincerely acknowledge the generous gifts of fly stocks from the respective sources. For most of the immunostaining and analysis, 2-day old adult males grown at room temperature were used. The conditional mutant alleles of *shibire/dynamin *(*shi *^*ts1 *^and *shi *^*ts2 *^) were grown at 18°C and 2-day old adults were shifted to either 29°C or 32°C for a defined period before dissection and immunostaining. All of the transgenic fly stocks were grown at 25°C. The crosses set for the heat pulse studies were grown at 18°C. Subsequently, the freshly emerged males were maintained at the same temperature for 2 days before they were shifted to the non-permissive temperatures as per the requirements.

### Immunohistochemistry

For whole mount analysis, testes were dissected in phosphate buffered saline (PBS) [69] containing 0.01% saponin (Sigma Chemical Co., MO, USA), incubated in the same solution for 30 minutes and then fixed in PBS containing 4% paraformaldehyde (freshly prepared). After several quick rinses in PTX (PBS with 0.3% Triton X-100), the fixed testes were incubated in different primary antibody solutions in PTX for 1 hour at room temperature. This was followed by washes in PTX, and further incubation in appropriate fluorescent labeled secondary antisera in PTX for 1 hour. After a final series of washes, the tissue preparations were mounted on a glass slide with a drop of Vectashield^® ^(Vector Laboratories Inc., USA) and under a #1 cover glass. To observe the nuclei and the F-actin distribution in the tissue, the immunostained specimen were additionally incubated in PTX containing 76 μM of FITC:phalloidin or RITC:phalloidin (Sigma Chemical Co., MO, USA) and 0.001% DAPI (Sigma Chemical Co., MO, USA) for 30 minutes, washed in PTX and then mounted as above. In some cases individual cysts were teased out of the testis after staining and dispersed on the slide before mounting. This helped to reveal the subcellular distribution of F-actin structures. For squash preparations, the testes were dissected on a plus charged slide, squashed under a cover slip, dipped in liquid nitrogen for a few minutes and then in ice-cold 95% ethanol for 3 minutes, or, until the cover slip dropped off. This was followed by a post fixation of the slide in PBS containing 4% paraformaldehyde and immunostaining as per the procedure described earlier [46]. This often disrupted the head cyst cells but kept the NBs intact. In all of these cases the actin caps were found to remain associated with the intact NBs.

For the pharmacological treatments, adult testes were dissected in PBS containing 0.003% DMSO and either 30 μM lat B or 5 μM vinb (both from Sigma Chemical Co., MO, USA), and then incubated in the same media for 30 minutes before fixation. Then they were processed as described above. For short heat pulses, dissected testes in PBS were incubated in a water bath set at defined temperatures (18°C for controls and 29°C for heat pulse) for the specified period of time. Then they were processed as described above.

A list of all of the antibodies used for this study and their respective sources is provided in Additional file 7. All of the fluorescently conjugated secondary antibodies were obtained from the Jackson Research Laboratories Inc., USA, and from the Molecular Probes Inc., OR, USA, and used at 1:400 dilutions.

### Electron microscopy

Testes were dissected from 2-day old males in 2.5% glutaraldehyde (EM Sciences Inc., USA), 4% paraformaldehyde and 0.04% CaCl_2 _in 0.1 M phosphate buffer (pH 7.4) at 4°C, then fixed overnight in the same solution, washed in 0.1 M phosphate buffer (pH 7.4) and post-fixed in OsO_4 _for 4 hours at 4°C. This was followed by several washes in 0.1 M phosphate buffer (pH 7.4), dehydration in graded series of ethanol, and embedding in Araldite (E. Merk GmbH, Germany). Ultrathin (100 nm) sections were obtained in Leica Ultracut 6b, stained with aqueous uranyl acetate and lead citrate, and imaged in a JEOL 100S electron microscope as per the procedure described previously [46].

### Statistical analyses of the NB and IC morphologies in the wild type and mutants

The number of mature NBs and the ICs were counted in each testis preparation under a 40 × 0.75 NA objective fitted in an epifluorescence microscope. The NBs and the ICs were carefully scrutinized for abnormal organizations and if they were found to be out of register with each other, then they were counted as disrupted. The principal criteria used for this analysis has been described earlier [8]. Briefly, the criterion for a NB to be considered as intact, most of the nuclei should remain in parallel register and be packed tightly together (example: Figure 6C, a). Only those which appeared obviously disrupted were counted as not intact (example: Figure 6C, b and 6C, c). The F-actin cones in an IC are found in a parallel register (example: Figure 6E, a) while the disrupted ones had them scattered to different extents (example: Figure 6E, b and 6E, c). Volunteers also counted some of the preps, selected at random, in a double blind manner. This showed that the criteria used for counting were quite robust. The results were presented as histogram plots with ± standard error of the mean, and the significance of the differences was calculated by using the Mann-Whitney non-parametric test using the Graphpad Instat™ software.

### Correlation analysis

The NB and IC defects as well as the total numbers were recorded from 10 lat B treated testes. The relative NB and IC disruption values from individual testis were plotted on the *x *- and *y *-axis, respectively. The data was then analyzed by using Graphpad Instat™ and the SigmaPlot™. Graphpad Instat™ was used to determine the pairwise significance (*p *) values and to estimate whether the slope was significantly different from zero. SigmaPlot™ was used to determine the slope of the best-fitted line amongst the points in the plot. For studying the correlation between different genotypes, the average NB and IC disruption values (determined as a percentage of the total) from each genotype were plotted and analyzed as described above.

### Image collection and analysis

All images were collected by using the Olympus FV1000SPD laser scanning confocal microscope (LSCM). The image frames were merged by using ImageJ^® ^, and adjusted for their brightness and contrast to maintain uniform visibility in a montage by using Adobe Photoshop^® ^(Adobe Corp., USA). The figures were then organized and labeled in Adobe Illustrator™.

## Abbreviations

AJ: adherence junction; ck: crinkled; clc: clathrin light chain; DAPI: 4',6-diamidino-2-phenylindole; DIC: differential interference contrast; ER: endoplasmic reticulum; ES: ectoplasmic specialization; FITC: fluorescein isothiocyanate; GFP: green fluorescent protein; IC: individualization complex; jar: jaguar; lat B: latrunculin B; mRFP1: monomeric red fluorescent protein; NB: nuclei bundle of maturing spermatids; RITC: rhodamine isothiocyanate; shi: shibire; sqh: spaghetti-squash; SV: seminal vesicle; TEM: transmission electron microscopy; UAS: upstream activating sequence; vinb: vinbalstine; WASP: Wiskott-Aldrich syndrome protein.

## Authors' contributions

BSD made the initial observation, designed and executed the majority of the experiments, analyzed the data, composed the figures and helped in writing the manuscript. KR participated in the experimental design in collaboration with BSD and executed the final set of experiments, analyzed the data, composed the figures and the manuscript, and supervised the whole project. SS performed the electron microscopy of wild-type testis and provided the TEM data.

## Supplementary Material

Additional file 1 **A Three-dimensional rocking movie of the nuclei bundles and actin caps distribution at the base of the wild-type testes**. A confocal Z-focus series of an immunostained specimen was collected by using a 40× NA 0.9 objective. The testes preparation was mounted with protective spacers to maintain the original spatial distribution in the tissue. Nuclei were stained with 4',6-diamidino-2-phenylindole (DAPI) (blue) and F-actin was stained with fluorescein isothiocyanate (FITC):phalloidin (green). A corresponding Z-projection of the image stack is shown in Figure 1(A). Click here for file

Additional file 2 **Movie of the acrosome movements in the wild-type testis in phosphate buffered saline**. The time-lapsed images of isolated *y w; P [w *^+*mc *^*snky:GFP] *testis collected using a 60× (oil) NA 1.42 objective attached to a laser scanning confocal microscope. Each frame was averaged for 2 scans (at around 1 second per scan) and collected every 3 minutes. A total of 20 frames are presented in this movie. The movie runs at two frames per second. The acrosomes of the elongating spermatids (red arrows) were found to move quite well. In comparison, the compacted acrosome bundles were mostly stationary (white arrows). A two-dimensional time projection of these frames is shown in Figure 5(A). Click here for file

Additional file 3 **The Gal4/UAS-reporter expression patterns in the testes**. (A) Confocal sections show myosin VII-GFP (green) and myr-mRFP1 localizations in the testis from the *w UAS-myosin VII-GFP pCOGGal4/Y; UAS-myr-mRFP1/+ *adults. The expression was localized in the germ-line stem cells and primary gonial precursors (arrowheads) at an early stage. Later on, it was contained in the cyst cells (arrows). The *UAS-myr-mRFP1 *(myristoylated mRFP1) expression generally marks the cell membrane and highlights the cysts cell perimeter (arrows, A") around the spermatocytes in the testis. (B) The UAS-actin:GFP expression in the *pCOGGal4/Y; UAS-actin:GFP/+ *testis is increased in the head cyst cells (HC, dotted lines) at the final stages before the mature sperm release from the cysts. B-B" indicates different focus levels of the same testis. The rhodamine isothiocyanate (RITC):phalloidin (red) and 4',6-diamidino-2-phenylindole (DAPI) (blue) staining are shown in appropriate false colors. (C) The head cyst cells (arrows) are prominently marked by the combined expression of *UAS-GFP *and *UAS-actin:GFP *in *SG18.1Gal4 *background during the sperm individualization stages. Click here for file

Additional file 4 **Movie of the acrosome movements in the wild type testis in phosphate buffered saline containing 5 μM vinblastine**. The time-lapse images of isolated *y w; P [w *^+*mc *^*snky:GFP] *testis collected using a 60× (oil) NA 1.42 objective attached to a laser scanning confocal microscope as described for Additional file 5. The movie runs at two frames per second. The testes preparations were treated for 30 minutes in phosphate buffered saline containing 5 μM vinb before imaging. A total of 10 frames are presented in this movie with each frame having its own time stamp. The acrosomes of the elongating spermatids (red arrows) as well as the compacted acrosome bundles (white arrows) appeared to move very slowly. The two-dimensional time projection of these frames is shown in Figure 5(B). Click here for file

Additional file 5 **Transient disruption of the *shibire *function in the head cyst cell caused actin cap and NB disruptions**. The *UAS-shi *^*ts1 *^expression in the head cyst cells due to the (A) *pCOGGal4 *and (B) *SG18.1Gal4 *drivers, disrupted the actin caps and nuclei bundles (NBs) at non-permissive temperature (29°C). Rhodamine isothiocyanate (RITC):phalloidin and 4',6-diamidino-2-phenylindole (DAPI) staining of these testes before (a), (b) and after (c), (d) the heat pulse showed visible disruptions of the actin caps and NBs (arrows). In addition, there was punctate accumulation of F-actin in the head cyst cell cytoplasm (arrowheads) after the heat pulse. Fine arrows indicate mature nuclei separated from the actin caps. This is not found in the wild-type controls treated in a similar manner. The actin cap and the NB morphology remained unaltered even after 30 minutes at 29°C in the *pCOGGal4 UAS-GFPck/Y *and *SG18.1Gal4 UAS-GFP/UAS-actin:GFP *testes (data not shown). (C) The *UAS-WASP *^*ΔCA *^expressions by using (a), (b) *pCOGGal4 *and (c)-(e) *SG18.1Gal4 *caused mild loss of F-actin staining from the actin caps but no detectable NB disruptions even after a 30 minute heat pulse. Click here for file

Additional file 6 **Table S1**. Table listing *Drosophila melanogaster *stocks used in this study Click here for file

Additional file 7 **Table S2**. Table listing the antibodies used in this study Click here for file

## References

[B1] Lindsley DL, Tokuyasu KT, Ashburner M, Wright TRF (1980). Spermatogenesis. The Genetics and Biology of Drosophila.

[B2] Fuller MT (1998). Genetic control of cell proliferation and differentiation in *Drosophila *spermatogenesis. Semin Cell Dev Biol.

[B3] Tokuyasu KT, Peacock WJ, Hardy RW (1972). Dynamics of spermiogenesis in *Drosophila melanogaster *. I. Individualization process. Z Zellforsch Mikrosk Anat.

[B4] Tokuyasu KT, Peacock WJ, Hardy RW (1972). Dynamics of spermiogenesis in *Drosophila melanogaster *. II. Coiling process. Z Zellforsch Mikrosk Anat.

[B5] Noguchi T, Miller KG (2003). A role for actin dynamics in individualization during spermatogenesis in *Drosophila melanogaster *. Development.

[B6] Rogat AD, Miller KG (2002). A role for myosin VI in actin dynamics at sites of membrane remodeling during *Drosophila *spermatogenesis. J Cell Sci.

[B7] Frank DJ, Noguchi T, Miller KG (2004). Myosin VI: a structural role in actin organization important for protein and organelle localization and trafficking. Curr Opin Cell Biol.

[B8] Ghosh-Roy A, Desai BS, Ray K (2005). Dynein light chain 1 regulates dynamin-mediated F-actin assembly during sperm individualization in *Drosophila *. Mol Biol Cell.

[B9] Mermall V, Bonafe N, Jones L, Sellers JR, Cooley L, Mooseker MS (2005). *Drosophila *myosin V is required for larval development and spermatid individualization. Dev Biol.

[B10] Arama E, Agapite J, Steller H (2003). Caspase activity and a specific cytochrome C are required for sperm differentiation in *Drosophila *. Dev Cell.

[B11] Huh JR, Vernooy SY, Yu H, Yan N, Shi Y, Guo M, Hay BA (2004). Multiple apoptotic caspase cascades are required in nonapoptotic roles for *Drosophila *spermatid individualization. PLoS Biol.

[B12] Russell LD, Peterson RN (1985). Sertoli cell junctions: morphological and functional correlates. Int Rev Cytol.

[B13] Mruk DD, Cheng CY (2004). Cell-cell interactions at the ectoplasmic specialization in the testis. Trends Endocrinol Metab.

[B14] Bannister LH, Dyson M, Williams PL (1995). Reproductive system. Gray's Anatomy.

[B15] Cheng CY, Mruk DD (2002). Cell junction dynamics in the testis: Sertoli-germ cell interactions and male contraceptive development. Physiol Rev.

[B16] Wang CQ, Cheng CY (2007). A seamless trespass: germ cell migration across the seminiferous epithelium during spermatogenesis. J Cell Biol.

[B17] Mulholland DJ, Dedhar S, Vogl AW (2001). Rat seminiferous epithelium contains a unique junction (ectoplasmic specialization) with signaling properties both of cell/cell and cell/matrix junctions. Biol Reprod.

[B18] Aumuller G, Seitz J (1988). Immunocytochemical localization of actin and tubulin in rat testis and spermatozoa. Histochemistry.

[B19] Lee NP, Cheng CY (2004). Adaptors, junction dynamics, and spermatogenesis. Biol Reprod.

[B20] Wilson KL, Fitch KR, Bafus BT, Wakimoto BT (2006). Sperm plasma membrane breakdown during *Drosophila *fertilization requires sneaky, an acrosomal membrane protein. Development.

[B21] Lecuit T (2005). Adhesion remodeling underlying tissue morphogenesis. Trends Cell Biol.

[B22] Kuznetsov G, Bush KT, Zhang PL, Nigam SK (1996). Perturbations in maturation of secretory proteins and their association with endoplasmic reticulum chaperones in a cell culture model for epithelial ischemia. Proc Natl Acad Sci USA.

[B23] Mazzarella RA, Srinivasan M, Haugejorden SM, Green M (1990). ERp72, an abundant luminal endoplasmic reticulum protein, contains three copies of the active site sequences of protein disulfide isomerase. J Biol Chem.

[B24] Qualmann B, Kelly RB (2000). Syndapin isoforms participate in receptor-mediated endocytosis and actin organization. J Cell Biol.

[B25] Kay JG, Murray RZ, Pagan JK, Stow JL (2006). Cytokine secretion via cholesterol-rich lipid raft-associated SNAREs at the phagocytic cup. J Biol Chem.

[B26] Faix J, Rottner K (2006). The making of filopodia. Curr Opin Cell Biol.

[B27] Schober JM, Komarova YA, Chaga OY, Akhmanova A, Borisy GG (2007). Microtubule-targeting-dependent reorganization of filopodia. J Cell Sci.

[B28] Schulze KL, Bellen HJ (1996). *Drosophila *syntaxin is required for cell viability and may function in membrane formation and stabilization. Genetics.

[B29] Niessen CM (2007). Tight junctions/adherens junctions: basic structure and function. J Invest Dermatol.

[B30] Petit C (2001). Usher syndrome: from genetics to pathogenesis. Annu Rev Genomics Hum Genet.

[B31] Berg JS, Derfler BH, Pennisi CM, Corey DP, Cheney RE (2000). Myosin-X, a novel myosin with pleckstrin homology domains, associates with regions of dynamic actin. J Cell Sci.

[B32] Bohil AB, Robertson BW, Cheney RE (2006). Myosin-X is a molecular motor that functions in filopodia formation. Proc Natl Acad Sci USA.

[B33] Buss F, Spudich G, Kendrick-Jones J (2004). Myosin VI: cellular functions and motor properties. Annu Rev Cell Dev Biol.

[B34] Edwards KA, Kiehart DP (1996). *Drosophila *nonmuscle myosin II has multiple essential roles in imaginal disc and egg chamber morphogenesis. Development.

[B35] Hasson T (1997). Unconventional myosins, the basis for deafness in mouse and man. Am J Hum Genet.

[B36] Geisbrecht ER, Montell DJ (2002). Myosin VI is required for E-cadherin-mediated border cell migration. Nat Cell Biol.

[B37] Kiehart DP, Franke JD, Chee MK, Montague RA, Chen TL, Roote J, Ashburner M (2004). *Drosophila *crinkled, mutations of which disrupt morphogenesis and cause lethality, encodes fly myosin VIIA. Genetics.

[B38] Brodsky FM, Chen CY, Knuehl C, Towler MC, Wakeham DE (2001). Biological basket weaving: formation and function of clathrin-coated vesicles. Annu Rev Cell Dev Biol.

[B39] Williamson T, Gordon-Weeks PR, Schachner M, Taylor J (1996). Microtubule reorganization is obligatory for growth cone turning. Proc Natl Acad Sci USA.

[B40] Gordon-Weeks PR (2004). Microtubules and growth cone function. J Neurobiol.

[B41] Lan Y, Papoian GA (2008). The stochastic dynamics of filopodial growth. Biophys J.

[B42] Fenteany G, Zhu S (2003). Small-molecule inhibitors of actin dynamics and cell motility. Curr Top Med Chem.

[B43] Jordan MA, Wilson L (2004). Microtubules as a target for anticancer drugs. Nat Rev Cancer.

[B44] Gvakharia BO, Bebas P, Cymborowski B, Giebultowicz JM (2003). Disruption of sperm release from insect testes by cytochalasin and beta-actin mRNA mediated interference. Cell Mol Life Sci.

[B45] Bliek AM van der, Meyerowitz EM (1991). Dynamin-like protein encoded by the *Drosophila *shibire gene associated with vesicular traffic. Nature.

[B46] Ghosh-Roy A, Kulkarni M, Kumar V, Shirolikar S, Ray K (2004). Cytoplasmic dynein-dynactin complex is required for spermatid growth but not axoneme assembly in *Drosophila *. Mol Biol Cell.

[B47] Estes PS, Roos J, Bliek A van der, Kelly RB, Krishnan KS, Ramaswami M (1996). Traffic of dynamin within individual *Drosophila *synaptic boutons relative to compartment-specific markers. J Neurosci.

[B48] Chen ML, Green D, Liu L, Lam YC, Mukai L, Rao S, Ramagiri S, Krishnan KS, Engel JE, Lin JJ, Wu CF (2002). Unique biochemical and behavioral alterations in *Drosophila *shibire(ts1) mutants imply a conformational state affecting dynamin subcellular distribution and synaptic vesicle cycling. J Neurobiol.

[B49] Tal T, Vaizel-Ohayon D, Schejter ED (2002). Conserved interactions with cytoskeletal but not signaling elements are an essential aspect of *Drosophila *WASp function. Dev Biol.

[B50] Schafer G, Weber S, Holz A, Bogdan S, Schumacher S, Muller A, Renkawitz-Pohl R, Onel SF (2007). The Wiskott-Aldrich syndrome protein (WASP) is essential for myoblast fusion in *Drosophila *. Dev Biol.

[B51] Virkki N (1969). Sperm bundles and phylogenesis. Z Zellforsch Mikrosk Anat.

[B52] Åbro A (1998). Structure and development of sperm bundles in the dragonfly *Aeshna juncea L*. (Odonata). J Morphol.

[B53] Shen L, Turner JR (2005). Actin depolymerization disrupts tight junctions via caveolae-mediated endocytosis. Mol Biol Cell.

[B54] Russell LD (1980). Sertoli-germ cell interrelations: A review. Gamete Res.

[B55] Guttman JA, Takai Y, Vogl AW (2004). Evidence that tubulobulbar complexes in the seminiferous epithelium are involved with internalization of adhesion junctions. Biol Reprod.

[B56] Maniak M (2001). Cell adhesion: ushering in a new understanding of myosin VII. Curr Biol.

[B57] Mege RM, Gavard J, Lambert M (2006). Regulation of cell-cell junctions by the cytoskeleton. Curr Opin Cell Biol.

[B58] Chhabra ES, Higgs HN (2007). The many faces of actin: matching assembly factors with cellular structures. Nat Cell Biol.

[B59] Fulga TA, Rorth P (2002). Invasive cell migration is initiated by guided growth of long cellular extensions. Nat Cell Biol.

[B60] McNiven MA, Kim L, Krueger EW, Orth JD, Cao H, Wong TW (2000). Regulated interactions between dynamin and the actin-binding protein cortactin modulate cell shape. J Cell Biol.

[B61] Orth JD, Krueger EW, Cao H, McNiven MA (2002). The large GTPase dynamin regulates actin comet formation and movement in living cells. Proc Natl Acad Sci USA.

[B62] McNiven MA, Baldassarre M, Buccione R (2004). The role of dynamin in the assembly and function of podosomes and invadopodia. Front Biosci.

[B63] Orth JD, Krueger EW, Weller SG, McNiven MA (2006). A novel endocytic mechanism of epidermal growth factor receptor sequestration and internalization. Cancer Res.

[B64] Lie PP, Xia W, Wang CQ, Mruk DD, Yan HH, Wong CH, Lee WM, Cheng CY (2006). Dynamin II interacts with the cadherin- and occludin-based protein complexes at the blood-testis barrier in adult rat testes. J Endocrinol.

[B65] Vaid KS, Guttman JA, Babyak N, Deng W, McNiven MA, Mochizuki N, Finlay BB, Vogl AW (2007). The role of dynamin 3 in the testis. J Cell Physiol.

[B66] Parreira GG, Melo RC, Russell LD (2002). Relationship of sertoli-sertoli tight junctions to ectoplasmic specialization in conventional and en face views. Biol Reprod.

[B67] Beardsley A, O'Donnell L (2003). Characterization of normal spermiation and spermiation failure induced by hormone suppression in adult rats. Biol Reprod.

[B68] Kusumi N, Watanabe M, Yamada H, Li SA, Kashiwakura Y, Matsukawa T, Nagai A, Nasu Y, Kumon H, Takei K (2007). Implication of amphiphysin 1 and dynamin 2 in tubulobulbar complex formation and spermatid release. Cell Struct Funct.

[B69] Sambrook J, Fritsch EF, Maniatis T (1989). Molecular Cloning. A Laboratory Manual.

