# Supplementary material for: F-actin-based extensions of the head cyst cell adhere to the maturing spermatids to maintain them in a tight bundle and prevent their premature release in Drosophila testis
Source: BMC Biol. 2009 May 5;7:19. doi: 10.1186/1741-7007-7-19 (PMC2683793; doi:10.1186/1741-7007-7-19)

Supplemental Figure 1: Gal4 expression patterns in the *Drosophila* testis.

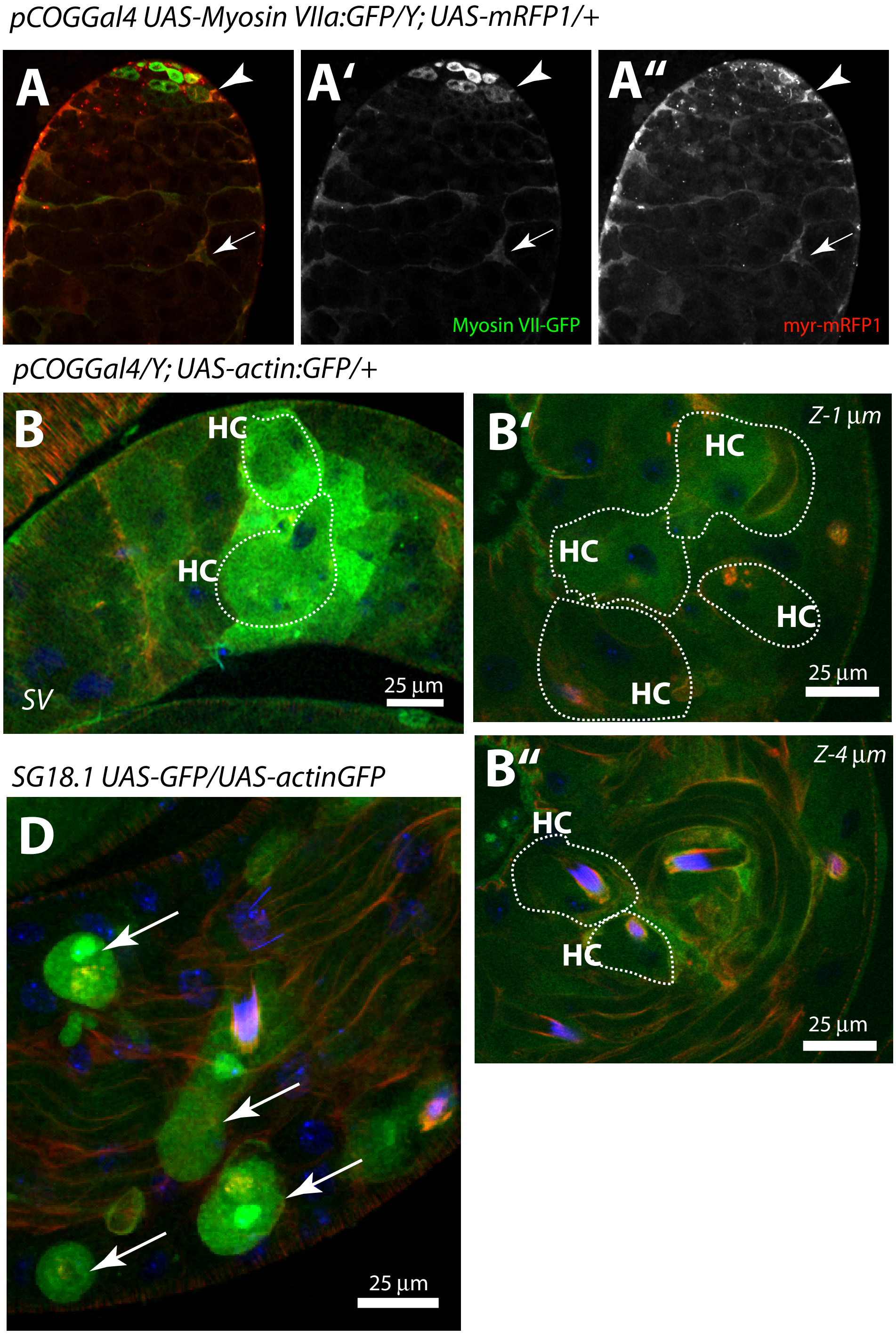

Supplement: Additional file 3 — The Gal4/UAS-reporter expression patterns in the testes. (A) Confocal sections show myosin VII-GFP (green) and myr-mRFP1 localizations in the testis from the w UAS-myosin VII-GFP pCOGGal4/Y; UAS-myr-mRFP1/+ adults. The expression was localized in the germ-line stem cells and primary gonial precursors (arrowheads) at an early stage. Later on, it was contained in the cyst cells (arrows). The UAS-myr-mRFP1 (myristoylated mRFP1) expression generally marks the cell membrane and highlights the cysts cell perimeter (arrows, A") around the spermatocytes in the testis. (B) The UAS-actin:GFP expression in the pCOGGal4/Y; UAS-actin:GFP/+ testis is increased in the head cyst cells (HC, dotted lines) at the final stages before the mature sperm release from the cysts. B-B" indicates different focus levels of the same testis. The rhodamine isothiocyanate (RITC):phalloidin (red) and 4',6-diamidino-2-phenylindole (DAPI) (blue) staining are shown in appropriate false colors. (C) The head cyst cells (arrows) are prominently marked by the combined expression of UAS-GFP and UAS-actin:GFP in SG18.1Gal4 background during the sperm individualization stages. [file 1741-7007-7-19-S3.pdf]
