# Supplementary material for: F-actin-based extensions of the head cyst cell adhere to the maturing spermatids to maintain them in a tight bundle and prevent their premature release in Drosophila testis
Source: BMC Biol. 2009 May 5;7:19. doi: 10.1186/1741-7007-7-19 (PMC2683793; doi:10.1186/1741-7007-7-19)
Supplement: Additional file 6 — Table S1. Table listing Drosophila melanogaster stocks used in this study [file 1741-7007-7-19-S6.doc]

**Additional Table 1: List of *Drosophila melanogaster*** stocks used in this study

| Fly Stocks used | Nature | Source | Reference/Source |
| --- | --- | --- | --- |
| *y w; P[w^+mc^ snky-GFP]* | Transgene, recombinant Sneaky-GFP fusion protein under native promoter | B. Wakimoto, Washington, USA. | Wilson *et al.*, 2006 |
| *sqh^AX3^; P[w+ sqh-gfp]42* | Transgene, recombinant Sqh-GFP fusion protein under native promoter | A. Brand, Cambridge, UK | Royou *et al.*, 2002 |
| *w; actin5cGal4 UAS clc–GFP* | Transgene, recombinant CLC-GFP fusion protein under UAS promoter driven by actin5cGal4. | A. Majumdar, TIFR, India. | Chang *et al.,* 2002. |
| *w; UAS-GFP -myosin VII/ck* | Transgene, recombinant myosin VIIa-GFP fusion protein under UAS promoter | D. Kiehart, Duke Univ., NC, USA. | Todi *et al.*, 2005 |
| *w pCOGGal4* | Transgene, recombinant Gal4 under *otu* promoter | K. Miller, St Louis, USA. | Fulga and Rorth, 2002 |
| *w; sg18.1Gal4* | Transgene, recombinant Gal4, enhancer trap | V. Rodrigues, TIFR, India | Shyamala and Chopra, 1999 |
| *shi^ts1^* | EMS induced, temperature sensitive (*ts*), paralytic | Fly Stock Center, Bloomington, Indiana | van der Bliek and Meyerowitz, 1991 |
| *w: UAS-WASP^ΔCA^* | Transgene | Eyal Schejter | Tan et al., 2002 |
| *w; UAS-GFP* | Transgene | Fly Stock Center, Bloomington, Indiana | Dickson, 1996.3.29, [FBrf0086268] (www.flybase.org) |
| *w: UAS-myr-mRFP* | Transgene | -do- | Chang, 2003.6, [[FBrf0159887]](http://flybase.org/reports/FBrf0159887.html)  (www.flybase.org) |
