# Supplementary material for: F-actin-based extensions of the head cyst cell adhere to the maturing spermatids to maintain them in a tight bundle and prevent their premature release in Drosophila testis
Source: BMC Biol. 2009 May 5;7:19. doi: 10.1186/1741-7007-7-19 (PMC2683793; doi:10.1186/1741-7007-7-19)
Supplement: Additional file 7 — Table S2. Table listing the antibodies used in this study [file 1741-7007-7-19-S7.doc]

Additional Table 2: List of antibodies used in this study

| Antibodies used | Source | Dilutions | Reference |
| --- | --- | --- | --- |
| *Antibodies that stained in the head cyst cells and the actin caps* | | | |
| Lamin DM0 | DSHB (ADL40) | 1:10 | *Not known* |
| myosin VIIA | Dan Kiehart | 1:200 | Kiehart et al., 2004 |
| myosin VI | Kathryn Miller | 1:50 | Kellerman and Miller, 1992 |
| Ras | Cell Signaling Technology, USA (Cat #3965) | 1:200 | Boguski and McCormick, 1993. |
| DE-Cadherin | DSHB (DCAD2) | 1:100 | Oda et al., 1994 |
| Crumbs | DSHB (cq4) | 1:100 | Tepass et al., 1990 |
| Armadillo | DSHB(N27A1) | 1:10 | Riggleman et al, 1990 |
| ERp72 | Calbiochem, USA | 1:200 | Kuznetsov et al., 1996 |
| β-Tubulin | DSHB (E7) | 1:50 | Chu and Klymkowsky,1989. |
| Dynamin | K.S. Krishnan | 1:200 | Estes et al.,1996 |
| Syndapin | Vimlesh Kumar | 1:200 | Kumar et al, 2009 |
| WASP | Eyal Schejter | 1:200 | Tal et al., 2002 |
| Syntaxin | DSHB (8c3) | 1:10 | Fujita et al, 1992 |
| *Antibodies that did not stain in the head cyst cells and the actin caps* | | | |
| Spectrin | DSHB (3A9) | 1:50 | Dubreuil et al., 1987 |
| Eya | DSHB (eya10h6) | 1:10 | Bonini et al, 1993 |
| v-Src | Sigma Chemical Co., MO, USA | 1: 200 | *Not known*  (Cat # S-1686) |
| Lamin C | DSHB (LC28.26) | 1:10 | Riemer et al., 1995 |
| DSHB - Developmental Studies Hybridoma Bank, USA | | | |
