# Supplementary material for: F-actin-based extensions of the head cyst cell adhere to the maturing spermatids to maintain them in a tight bundle and prevent their premature release in Drosophila testis
Source: BMC Biol. 2009 May 5;7:19. doi: 10.1186/1741-7007-7-19 (PMC2683793; doi:10.1186/1741-7007-7-19)
Supplement: Additional file 5 — Transient disruption of the shibire function in the head cyst cell caused actin cap and NB disruptions. The UAS-shi ts1 expression in the head cyst cells due to the (A) pCOGGal4 and (B) SG18.1Gal4 drivers, disrupted the actin caps and nuclei bundles (NBs) at non-permissive temperature (29°C). Rhodamine isothiocyanate (RITC):phalloidin and 4',6-diamidino-2-phenylindole (DAPI) staining of these testes before (a), (b) and after (c), (d) the heat pulse showed visible disruptions of the actin caps and NBs (arrows). In addition, there was punctate accumulation of F-actin in the head cyst cell cytoplasm (arrowheads) after the heat pulse. Fine arrows indicate mature nuclei separated from the actin caps. This is not found in the wild-type controls treated in a similar manner. The actin cap and the NB morphology remained unaltered even after 30 minutes at 29°C in the pCOGGal4 UAS-GFPck/Y and SG18.1Gal4 UAS-GFP/UAS-actin:GFP testes (data not shown). (C) The UAS-WASP ΔCA expressions by using (a), (b) pCOGGal4 and (c)-(e) SG18.1Gal4 caused mild loss of F-actin staining from the actin caps but no detectable NB disruptions even after a 30 minute heat pulse. [file 1741-7007-7-19-S5.pdf]

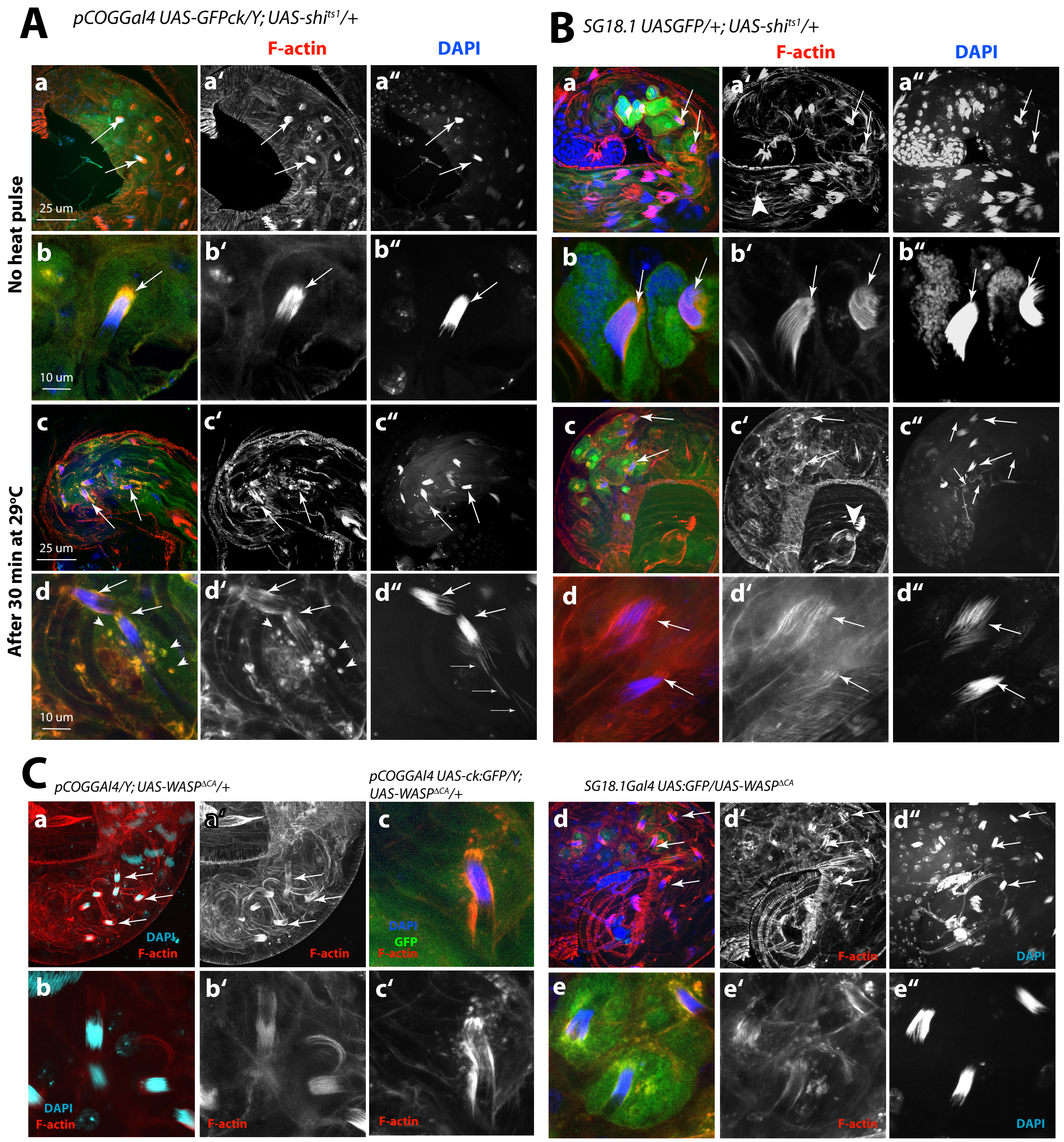

A
pCOGGal4 UAS-GFPck/Y; UAS-shi^{ts1}/+
F-actin
DAPI
No heat pulse
After 30 min at 29°C
25 um
10 um
25 um
10 um
B
SG18.1 UASGFP/+; UAS-shi^{ts1}/+
F-actin
DAPI
C
pCOGGAl4/Y; UAS-WASP$^{\Delta CA}$/+
pCOGGAl4 UAS-ck:GFP/Y; UAS-WASP$^{\Delta CA}$/+
SG18.1Gal4 UAS:GFP/UAS-WASP$^{\Delta CA}$
DAPI
F-actin
GFP
